# Mechanistic Insights into Redox-Dependent Macropinocytosis in Primary Human Neutrophils

**DOI:** 10.3390/antiox15070904

**Published:** 2026-07-21

**Authors:** Stephen A. Addo, Imre Babay, Amritha Sreekumar, Douglas Sloan, Tamasi Roy, Ananth Sudha, Jeffrey Thomas, Ryan A. Harris, Zoltán Benyó, Gábor Csányi

**Affiliations:** 1 Vascular Biology Center, Medical College of Georgia, Augusta University, 1460 Laney Walker Blvd., Augusta, GA 30912, USA; saddo@augusta.edu (S.A.A.); imibabay@gmail.com (I.B.); amsreekumar@augusta.edu (A.S.); dsloan2@augusta.edu (D.S.); taroy@augusta.edu (T.R.); sananth@augusta.edu (A.S.); 2 Institute of Clinical Pathophysiology, Semmelweis University, Tűzoltó utca 37-47, 1094 Budapest, Hungary; benyo.zoltan@semmelweis.hu; 3 Georgia Prevention Institute, Medical College of Georgia, Augusta University, 1499 Walton Wy, Augusta, GA 30912, USA; jethomas@augusta.edu (J.T.); ryharris@augusta.edu (R.A.H.); 4 HUN-REN-SU Cerebrovascular and Neurocognitive Disease Research Group, Tűzoltó utca 37-47, 1094 Budapest, Hungary; 5 Department of Pharmacology and Toxicology, Medical College of Georgia, Augusta University, 1460 Laney Walker Blvd., Augusta, GA 30912, USA

**Keywords:** macropinocytosis, neutrophils, NADPH oxidase, reactive oxygen species

## Abstract

Macropinocytosis is an actin-driven endocytic process mediating nonspecific uptake of extracellular fluid through membrane ruffling. Neutrophil macropinocytosis has been reported only in limited descriptive studies, and its signaling mechanisms have not been defined. Here, we provide a characterization of macropinocytosis in primary human neutrophils and investigate signaling pathways that contribute to the regulation of this process. Quantitative flow cytometry using a high-molecular-weight fluid-phase tracer showed that the diacylglycerol (DAG) mimetic 4β-phorbol 12-myristate 13-acetate (4β-PMA) induces macropinocytic activity in primary human neutrophils. Granulocyte macrophage-colony stimulating factor (GM-CSF) and hepatocyte growth factor (HGF) also promoted fluid-phase uptake. Inhibition of actin polymerization or macropinocytosis reduced tracer uptake, confirming dependence on actin-driven machinery. Scanning electron microscopy revealed dorsal membrane ruffling and cup-like structures after stimulation. Mechanistically, DAG-dependent activation of protein kinase C beta (PKCβ) acted upstream of NADPH oxidase 2 (NOX2)-derived superoxide anion production, driving membrane remodeling. Collectively, these findings define a DAG–PKCβ–NOX2–superoxide signaling axis, as a key regulator of macropinocytosis in primary human neutrophils and provide the first mechanistic framework for its redox-dependent regulation.

## 1. Introduction

Neutrophils are the most abundant circulating leukocytes and play a central role in innate immune defense. Upon recruitment to inflamed or infected tissues, they rapidly execute diverse effector functions, including phagocytosis, degranulation, reactive oxygen species (ROS) production, and neutrophil extracellular trap (NET) formation [1,2,3]. Within inflamed tissues and circulation, neutrophils are continuously exposed to complex extracellular cues including cytokines, lipid mediators, pathogen-associated signals, and cellular debris [4,5,6]. Efficient sampling of this extracellular environment is therefore essential for both systemic neutrophil activation and local sensing of interstitial inflammatory milieus enriched in soluble mediators and macromolecular cues.

Macropinocytosis is a highly conserved, actin-dependent endocytic process that mediates nonspecific uptake of extracellular fluid and soluble macromolecules through plasma membrane-derived vesicles known as macropinosomes [7,8,9]. This process is driven by dynamic membrane ruffling and actin remodeling that generate dorsal ruffles and macropinocytic cups, which internalize extracellular material into intracellular vesicles [10,11]. Macropinocytosis is strongly induced by growth factors, inflammatory mediators, and phorbol esters, all of which converge on cytoskeletal and membrane remodeling pathways [12,13]. Beyond its role in immune surveillance in macrophages and dendritic cells, macropinocytosis contributes to nutrient acquisition, metabolic adaptation, and environmental sensing, and its dysregulation is implicated in multiple disease states, including cancer, atherosclerosis, infectious diseases, chronic inflammatory disorders, and neurodegenerative diseases [14,15,16]. Despite these important roles in other systems, macropinocytosis in neutrophils remains largely unexplored, and its underlying signaling mechanisms have not been defined.

Importantly, neutrophil signaling is uniquely dominated by redox biology, as activation of the NADPH oxidase 2 (NOX2) complex generates large amounts of superoxide anion that function not only in microbial killing but also as spatially and temporally regulated signaling molecules. Reactive oxygen species (ROS) regulate phosphoinositide dynamics, actin remodeling, and membrane trafficking pathways that are fundamental to neutrophil function [17].

Here, we integrate quantitative flow cytometry and high-resolution imaging to characterize inducible macropinocytosis in primary human neutrophils and define the signaling mechanisms governing its activation. We show that diacylglycerol (DAG)–protein kinase C β (PKCβ) signaling activates a NOX2-dependent redox axis that drives neutrophil macropinocytosis. Using pharmacological, biochemical, and imaging approaches, we identify a previously unrecognized DAG–PKCβ–NOX2–ROS signaling pathway that orchestrates actin-dependent membrane ruffling and fluid-phase uptake in human neutrophils. These findings establish NOX2-derived redox signaling as a central and previously unrecognized regulator of neutrophil macropinocytosis, linking lipid second messengers to ROS-dependent membrane remodeling and endocytic activity.

## 2. Materials and Methods

### 2.1. Reagents

4β-phorbol 12-myristate 13-acetate (4β-PMA), 5-(N-ethyl-N-isopropyl) amiloride (EIPA), diphenyleneiodonium chloride (DPI), superoxide dismutase (SOD), catalase (CAT), sodium orthovanadate, 4-hydroxy-TEMPO (TEMPOL), cytochalasin d, calphostin c (CAL-C), ruboxistaurin hydrochloride (LY333531), rottlerin, and 1,2-dioctanoyl-sn-glycerol (DOG) were purchased from Sigma-Aldrich (St. Louis, MO, USA). 4α-phorbol 12-myristate 13-acetate (4α-PMA) was obtained from AdipoGen Corporation (San Diego, CA, USA). Fluorescein isothiocyanate (FITC)–dextran (70 kDa), Oregon Green dextran (70 kDa), and recombinant human proteins including CXCL12, platelet-derived growth factor (PDGF), epidermal growth factor (EGF), thrombospondin-1 (TSP1), and hepatocyte growth factor (HGF) were obtained from Thermo Fisher Scientific (Waltham, MA, USA). Anti-human monoclonal antibodies CD66b APC/Cyanine 7 and Siglec-8–PE/Cyanine 5 were purchased from BioLegend (San Diego, CA, USA). Human neutrophil isolation kit, recombinant human TNFα, and granulocyte–macrophage colony-stimulating factor (GM-CSF) were obtained from Miltenyi Biotec (San Diego, CA, USA). The small-molecule NADPH oxidase 2 (NOX2) inhibitor GSK2795039 was obtained from Tocris Bioscience (Bristol, UK). L-012 was purchased from Fujifilm (Tokyo, Japan). THP-1 monocytes were obtained from the American Type Culture Collection (ATCC; Manassas, VA, USA). TaqMan Reverse Transcription Kit and SYBR Green Super mix were purchased from Applied Biosystems (Grand Island, NY, USA). ViaDye Violet Fixable Viability Dye was purchased from Cytek Biosciences (Fremont, CA, USA).

### 2.2. Primary Human Neutrophil Isolation

Studies involving de-identified human blood samples provided by Dr. Ryan Harris (Georgia Prevention Institute, Augusta University, Augusta, GA, USA) were approved by the Augusta University Institutional Review Board (#1638053). Additional de-identified blood samples were obtained through the Shepeard Community Blood Center (Augusta, GA, USA) and handled under protocols approved by the Augusta University Institutional Biosafety Committee (#1458). Primary human neutrophils were isolated from anticoagulated whole blood by negative selection using the Miltenyi Biotec MACSxpress^®^ Neutrophil Isolation Kit (Miltenyi Biotec, Bergisch Gladbach, Germany) according to the manufacturer’s instructions. Briefly, whole blood was incubated with the isolation cocktail for 5 min at room temperature with gentle rotation, followed by magnetic separation using the MACSxpress^®^ Separator for 15 min. The magnetically labeled non-neutrophil cell fraction was retained, while the neutrophil-enriched supernatant was collected. Residual erythrocytes were removed by red blood cell lysis (ThermoFisher Scientific, Waltham, MA, USA), and purified neutrophils were washed and resuspended for downstream applications.

### 2.3. Flow Cytometry

Flow cytometry experiments were performed using the 4-Laser Acea Novocyte Quanteon flow cytometer (Agilent, Santa Clara, CA, USA). Human neutrophils were isolated from freshly collected anticoagulated whole blood using the MACSxpress^®^ Whole Blood Neutrophil Isolation Kit (Miltenyi Biotec) according to the manufacturer’s instructions through immunomagnetic negative selection. Residual erythrocytes were removed by red blood cell lysis prior to downstream analyses. Neutrophil purity and viability were assessed by flow cytometry using anti-CD66b to identify neutrophils and Siglec-8 to exclude eosinophil contamination. Live/dead discrimination (ViaDye Violet Fixable Viability Dye) was performed using a fixable viability dye, and purity was determined from the percentage of viable CD66b^+^ Siglec-8^−^ cells. A minimum of 10,000 events were collected for all experiments. Human neutrophils were treated with vehicle, PMA (1 μM) ± EIPA (25 μM, 30 min pre-incubation) in the presence of FITC-dextran (100 μg/mL, 60 min, 70 kDa, fluid-phase marker) or Oregon green dextran (50 μg/mL, 60 min, 70 kDa, fluid-phase marker). In separate experiments, neutrophils were treated with vehicle or PMA (1 μM, 60 min), in the absence or presence of calphostin c (CAL-C) (20 µM, 30 min pre-incubation), LY333531 (20 µM, 30 min pre-incubation), rottlerin (20 µM, 30 min pre-incubation), diphenyleneiodonium chloride (DPI) (5 μM, 30 min pre-incubation), MitoTempo (50 µM, 30 min pre-incubation), or GSK2795039 (5 μM, 30 min pre-incubation), and FITC-dextran uptake was quantified. Macropinocytic uptake of FITC-dextran and Oregon green dextran were quantified by measuring FITC fluorescence intensity (Ex: 488 nm, Em 530/30 nm).

### 2.4. Scanning Electron Microscopy

Human neutrophils were seeded onto human aortic endothelial cells (HAEC, ATCC). Endothelial cells were pretreated with recombinant human TNFα (10 ng/mL) for 4 h to induce adhesion molecule expression. Following adhesion, neutrophils were exposed to vehicle or 4β-PMA (1 μM, 20 min) in the presence or absence of 5-(N-ethyl-N-isopropyl) amiloride (EIPA, 25 μM, 30 min preincubation). Cells were fixed in 4% paraformaldehyde and 2% glutaraldehyde prepared in 0.1 M sodium cacodylate buffer at 4 °C for 16 h. Samples were subsequently dehydrated through a graded ethanol series (25–100%) and subjected to critical point drying (Tousimis Samdri-790, Rockville, MD, USA). Dried samples were sputter-coated with a 3.5 nm layer of gold–palladium (Anatek Hummer, Sparks, NV, USA) and imaged at 20 kV using a JEOL JSM-IT500HR InTouchScope scanning electron microscope (Tokyo, Japan). Neutrophil membrane ruffling was quantified and normalized to the number of cells within each field of view.

### 2.5. qRT-PCR

Total RNA was isolated from primary human neutrophils using an RNA extraction kit (IBI Scientific, Dubuque, IA, USA) according to the manufacturer’s protocol. The NanoDrop Microvolume Spectrophotometer (ThermoFisher Scientific) was used to determine RNA concentration and quality based on each sample’s optical density at 260 nm and 280 nm. The TaqMan Reverse Transcription Kit (Applied Biosystems, Grand Island, NY, USA) was used to generate complementary DNA from RNA. Real-time PCR was performed using the SYBR Green Supermix (Applied Biosystems). All amplifications were performed in triplicates and normalized to GAPDH. The Δ*C*t method was used to quantify relative gene expression. The primers used for real-time PCR are as follows:

*GAPDH*: F-CATGTTCGTCATGGGTGTGAACCA,

R-AGTGATGGCATGGACTGTGGTCAT,

*PKC epsilon*: F-GCCACTTCGAGGACTGGATTGAT,

R-TCACCCGACGACCCTGAGAGA,

*PKC gamma*: F-CCATCTGCAAGGGGTTCCTGA,

R-CCATGTGCACGGATGGTAGGTT,

*PKC alpha*: F-ATTCAAGCCCAAAGTGTGTGG,

R-GATCAGGTGGTGTTAAGACGGG,

*PKC eta*: F-CAGCCCACCTACTGCTCTCACT,

R-GATGATGGCAGCGTTTATGGA,

*PKC beta*: F-GACGTCCTCATTGTCCTCGTAAGA,

R-CTTTTGGGATCGGGAATCAGTT,

*PKC theta*: F-GAGGCTGTTAACCCTTACTGTGCTG,

R-GGATATACATCTGCCCGTTCTCTGA,

*PKC delta*: F-CAGCCTCAGGCCAAGGTGTT,

R-AGACTGTTTGCAATCCACGTCCT,

*PKC zeta*: F-GGCCACAGACTGGATTTTCT,

R-CTCGCTGGTGAACTGTGTGT,

*PKCBI*: F-AGCCAAAAGCTAGAGACAAGAGA,

R-GCACCGTGAATCCTGGAAGA,

*PKCBII*: F-TCTGCAAGGGCTGATGACC,

R-CCTGATGACTTCCTGGTCGG.

### 2.6. Quantification of Superoxide Anion Production Using L-012 Chemiluminescence

Superoxide production in primary human neutrophils was measured using the chemiluminescent probe L-012 (Fujifilm, Tokyo, Japan). Cells (5 × 10^4^ per well) were seeded in white, clear-bottom 96-well plates (Corning, Corning, NY, USA) in PBS containing L-012 (400 μM) and sodium orthovanadate (1 mM) (Sigma-Aldrich, St. Louis, MO, USA). Cells were pretreated with antioxidant enzymes SOD (Sigma-Aldrich) (150 U/mL, 15 min) or tempol (Sigma-Aldrich) (1 mM, 15 min), protein kinase c inhibitors (LY333531, rottlerin, or calphostin c) (Sigma-Aldrich) (20 µM, 15 min), pan-NOX inhibitor diphenyleneiodonium chloride (DPI) (Sigma-Aldrich) (5 μM, 15 min) or NOX2 inhibitor GSK2795039 (Tocris Bioscience) (20 μM, 30 min). Superoxide production was induced by stimulation with PMA (1 μM, 1 min), and chemiluminescence (relative light units, RLU) was recorded at 37 °C at 2 min intervals for 3 h using a microplate reader. Data were analyzed using MARS software V6.30 Edition 2 (BMG LABTECH, Ortenberg, Germany), and superoxide production was quantified as the area under the curve (AUC).

### 2.7. Statistical Analysis

All data are presented as means ± standard deviation (SD). The data were analyzed using GraphPad Prism 10.1.2 software (GraphPad Software Inc., Boston, MA, USA). Comparisons between groups were analyzed using a *t*-test or one/two-way analysis of variance (ANOVA) with Tukey’s post hoc test. *p* < 0.05 was defined as statistically significant.

## 3. Results

### 3.1. 4β-PMA Induces Actin-Dependent Macropinocytosis in Primary Human Neutrophils

**Figure 1:**
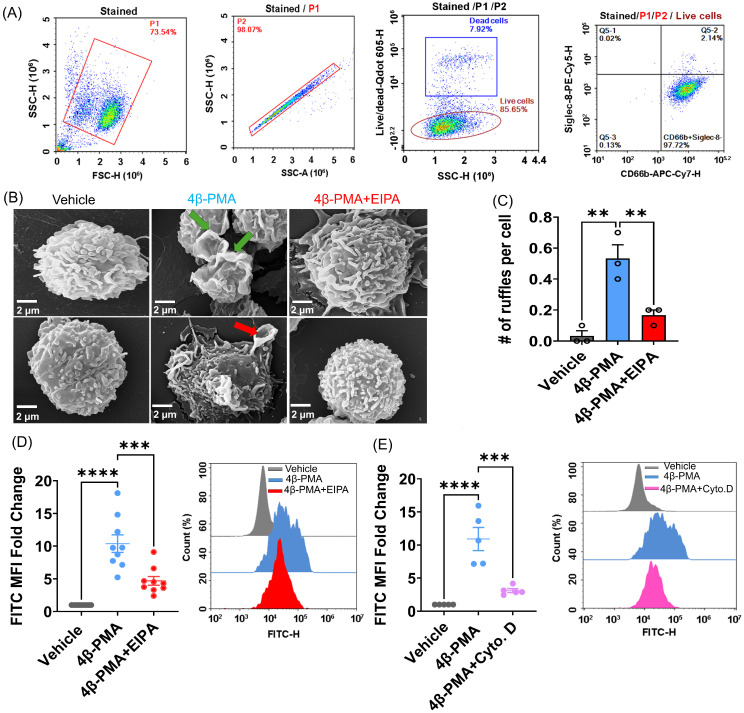
4β-PMA induces membrane ruffling and macropinocytosis in primary human neutrophils. (**A**) Representative flow cytometry gating strategy confirming isolation of a homogeneous CD66b^+^, Siglec-8^−^ neutrophil population from healthy donors prior to functional assays. (**B**) Primary human neutrophils adhered to human aortic endothelial cell (HAEC) monolayers were treated with vehicle, 4β-PMA (1 μM, 60 min), or 4β-PMA and EIPA (25 μM, 30 min pretreatment), followed by visualization of membrane ruffle formation using scanning electron microscopy. The upper and lower panels in each column show representative images from independent fields acquired under the indicated experimental condition. Green arrows indicate membrane ruffles reflecting active cytoskeletal remodeling, whereas red arrows indicate cup-shaped macropinocytic structures representing nascent macropinosome formation. Scale bars, 2 μm. (**C**) Quantification of membrane ruffling per cell under indicated conditions (*n* = 3). (**D**) Primary human neutrophils were incubated with FITC-dextran (70 kDa, 100 μg/mL) and stimulated with vehicle, 4β-PMA, or 4β-PMA and EIPA (*n* = 8). Dextran internalization was quantified by flow cytometry and expressed as fold change in mean fluorescence intensity (MFI). Representative histograms are shown. (**E**) Primary human neutrophils were treated with vehicle or 4β-PMA in the presence or absence of cytochalasin D (2 μM, 30 min), followed by assessment of FITC-dextran uptake by flow cytometry (*n* = 5). Data are presented as means ± SEM. Statistical significance was determined using one-way ANOVA with Tukey’s multiple-comparisons test. ** *p* < 0.01; *** *p* < 0.001; **** *p* < 0.0001.

Macropinocytic activity in primary human neutrophils remains poorly defined. To test whether neutrophils undergo inducible macropinocytosis, we stimulated cells with 4β-PMA, a gold-standard PKC activator and established inducer of macropinocytosis in monocytes and macrophages [18,19], and evaluated plasma membrane remodeling and functional responses. Primary human neutrophils were confirmed by flow cytometry as a CD66b^+^, Siglec-8^−^ population, consistent with a neutrophil identity and absence of eosinophil contamination (purity: [97 ± 1%], Figure 1A). Scanning electron microscopy showed that 4β-PMA (1 μM, 60 min) induced membrane ruffling and surface protrusions in neutrophils adherent to human aortic endothelial cell (HAEC) monolayers, used as a physiologically relevant endothelial substrate to enable stable adhesion and imaging of membrane dynamics. Quantification revealed an approximately 10-fold increase in membrane ruffling compared to vehicle controls (*p* < 0.05, Figure 1C). This response was significantly attenuated by pretreatment with the Na^+^/H^+^ exchange blocker EIPA (25 μM, 30 min), a canonical inhibitor of macropinocytosis. To assess macropinocytic internalization, uptake of FITC-dextran (70 kDa, 100 μg/mL) was measured by flow cytometry. 4β-PMA significantly increased dextran uptake in primary neutrophils (Figure 1D), which was strongly suppressed by EIPA, consistent with inhibition of macropinocytosis-dependent membrane dynamics. To confirm cytoskeletal dependence, neutrophils were pretreated with cytochalasin D prior to stimulation. Actin disruption reduced 4β-PMA-induced FITC-dextran uptake by [70%] (Figure 1E), confirming that internalization requires actin polymerization. Collectively, these findings demonstrate that 4β-PMA induces a coordinated program in primary human neutrophils characterized by actin-dependent remodeling, membrane ruffling, and fluid-phase uptake consistent with macropinocytosis.

### 3.2. Primary Human Neutrophils Display Time-Dependent and Stimulus-Specific Macropinocytic Activity

**Figure 2:**
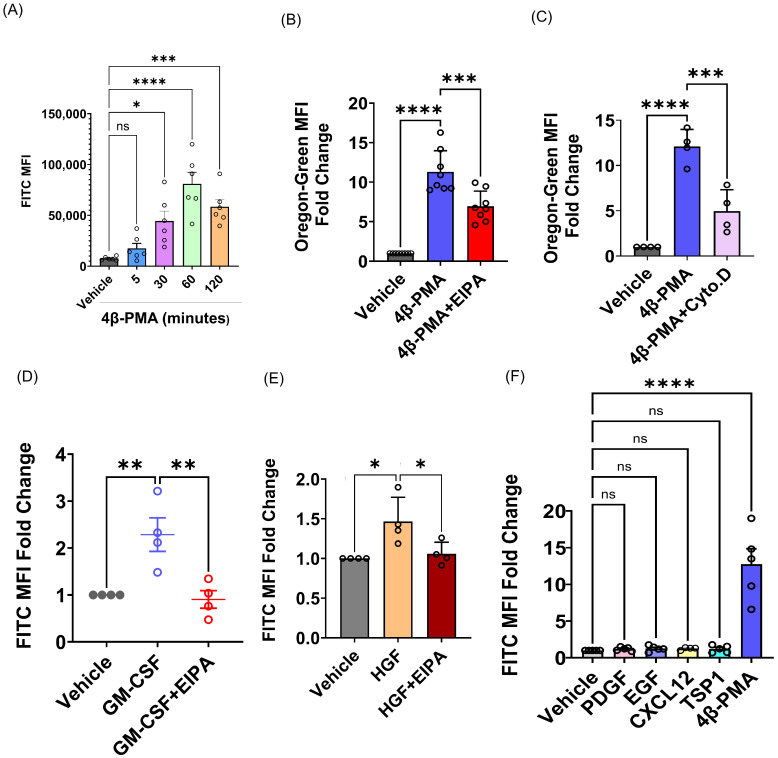
Primary human neutrophils show time-dependent and stimulus-specific macropinocytic responses. (**A**) Primary human neutrophils were incubated with FITC-dextran and stimulated with 4β-PMA. Dextran uptake was quantified over time by flow cytometry. (**B**,**C**) Neutrophils were stimulated with 4β-PMA in the presence or absence of EIPA or cytochalasin D and incubated with Oregon green dextran, a fluorophore with reduced pH sensitivity for assessment of fluid-phase uptake. Dextran internalization was quantified by flow cytometry. (**D**,**E**) Primary human neutrophils were incubated with FITC-dextran and stimulated with GM-CSF or HGF in the presence or absence of EIPA. Dextran uptake was quantified by flow cytometry. (**F**) Neutrophils were stimulated with PDGF, CXCL12, thrombospondin-1 (TSP1), or EGF in the presence of FITC-dextran. 4β-PMA was included as a positive control for macropinocytosis, and dextran uptake was quantified by flow cytometry. Data are presented as means ± SD. Statistical significance was determined using one-way or two-way ANOVA with Tukey’s multiple-comparisons test. ns, not significant; * *p* < 0.05; ** *p* < 0.01; *** *p* < 0.001; **** *p* < 0.0001.

Previous studies have shown that macropinocytosis stimulation exhibits distinct temporal kinetics in other cell types, with early membrane ruffling preceding maximal fluid-phase uptake [20,21]. Given the rapid and short-lived nature of neutrophil responses, we first defined the temporal profile of 4β-PMA-induced macropinocytic activity in primary human neutrophils. Human neutrophils were incubated with FITC-dextran (70 kDa, 100 μg/mL), stimulated with 4β-PMA, and fluid-phase uptake was quantified at defined time points by flow cytometry. 4β-PMA induced a progressive increase in dextran internalization, with a modest, significant increase at 30 min and a maximal response at 60 min, which then started to decline at 120 min, indicating that peak macropinocytic activity occurs within the first hour of stimulation (Figure 2A). Because FITC fluorescence is sensitive to vesicular acidification, we validated these findings using Oregon Green dextran, a fluorophore with substantially lower pH sensitivity. PMA significantly increased Oregon green dextran uptake, which was inhibited by both EIPA and cytochalasin D (Figure 2B,C). These findings confirm that the observed increase in fluorescence reflects genuine fluid-phase internalization and further demonstrate that macropinocytic uptake requires actin cytoskeletal remodeling. To determine whether physiologically relevant inflammatory mediators and growth factors stimulate macropinocytic activity in primary human neutrophils, we next examined the effects of multiple cytokines and growth factors previously implicated in endocytic and inflammatory signaling pathways. Neutrophils were incubated with FITC-dextran and stimulated with platelet-derived growth factor (PDGF), CXCL12, thrombospondin-1 (TSP1), epidermal growth factor (EGF), granulocyte macrophage-colony stimulating factor (GM-CSF), or hepatocyte growth factor (HGF). Among these, GM-CSF and HGF significantly increased dextran uptake (Figure 2D,E), whereas PDGF, CXCL12, TSP1, and EGF had no significant effect (Figure 2F).

Importantly, EIPA completely abolished GM-CSF- and HGF-induced dextran uptake, confirming that uptake occurs through macropinocytosis.

### 3.3. Stimulation of the DAG–PKCβ Signaling Axis Promotes Macropinocytosis in Primary Human Neutrophils

**Figure 3:**
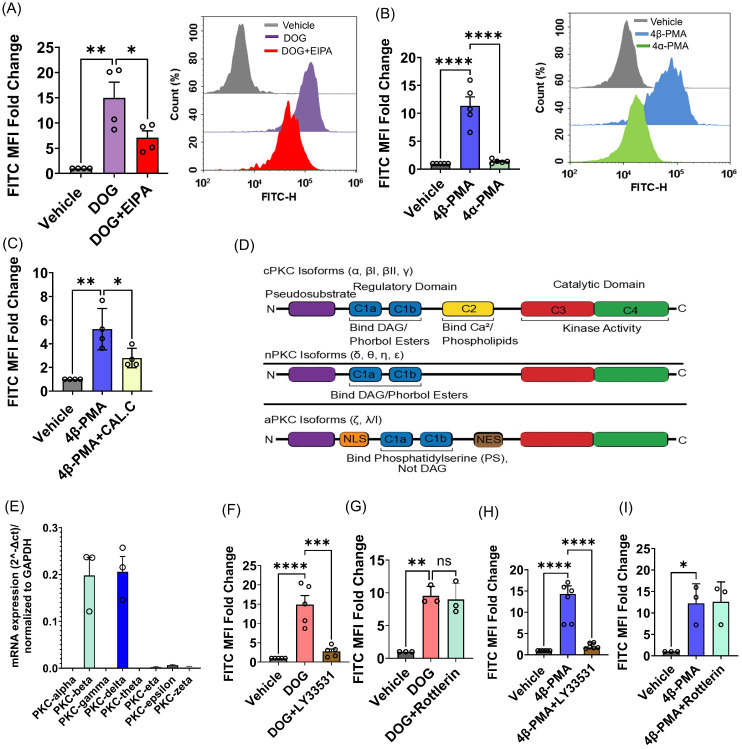
DAG–PKCβ signaling axis regulates macropinocytosis in primary human neutrophils. (**A**) Primary human neutrophils were stimulated with the DAG analog 1,2-dioctanoyl-sn-glycerol (DOG) (100 μM, 60 min) in the presence or absence of EIPA, followed by quantification of macropinocytic uptake of FITC-dextran by flow cytometry (*n* = 4). Representative histograms are shown. (**B**) Neutrophils were treated with vehicle, inactive 4α-PMA, or active 4β-PMA, followed by quantification of macropinocytic uptake of FITC-dextran by flow cytometry (*n* = 4). (**C**) Neutrophils were pretreated with the PKC inhibitor calphostin C (10 μM, 30 min) prior to 4β-PMA stimulation, followed by assessment of macropinocytic uptake of FITC-dextran by flow cytometry (*n* = 4). (**D**) Schematic overview of PKC isoform classification and activation requirements. (**E**) qPCR analysis of PKC isoform expression in primary human neutrophils (*n* = 3). (**F**,**G**) Neutrophils were stimulated with DOG in the presence or absence of the PKCβ-selective inhibitor LY333531 or the PKCδ inhibitor rottlerin (5 μM, 30 min), followed by quantification of macropinocytic uptake of FITC-dextran by flow cytometry. (**H**,**I**) Neutrophils were stimulated with 4β-PMA following pharmacological inhibition of PKCβ or PKCδ, and macropinocytic uptake of FITC-dextran was assessed by flow cytometry. Data are presented as means ± SD. Statistical significance was determined using one-way ANOVA with Tukey’s multiple-comparisons test. ns, not significant; * *p* < 0.05; ** *p* < 0.01; *** *p* < 0.001; **** *p* < 0.0001.

DAG is a central lipid second messenger generated downstream of receptor signaling that mediates activation of PKC [22,23]. Because phorbol esters, including PMA, function as stable DAG mimetics by binding the PKC C1 regulatory domain, we investigated whether DAG–PKC signaling regulates macropinocytosis in primary human neutrophils. To test this, cells were stimulated with the membrane-permeable DAG analog 1,2-dioctanoyl-sn-glycerol (DOG, 100 μM), and fluid-phase uptake was quantified by flow cytometry. DOG markedly increased dextran internalization compared with vehicle-treated controls (Figure 3A), accompanied by a clear rightward shift in fluorescence intensity. Importantly, EIPA pretreatment significantly reduced DOG-induced dextran uptake, indicating that DAG-dependent signaling promotes macropinocytic activity. Consistent with DAG mimetic signaling, stimulation with the active phorbol ester 4β-PMA induced a marked increase in FITC-dextran uptake, whereas the inactive stereoisomer 4α-PMA had no effect (Figure 3B). Furthermore, pharmacological inhibition of PKC with calphostin C (pan-PKC inhibitor) significantly attenuated 4β-PMA-induced dextran uptake (Figure 3C), demonstrating that PMA-driven macropinocytosis is PKC-dependent. PKC comprises a family of serine/threonine kinases classified into conventional, novel, and atypical isoforms based on cofactor requirements and structural features (Figure 3D). qPCR analysis revealed that PKCβ and PKCδ are the predominant PKC isoforms expressed in primary human neutrophils (Figure 3E). To further characterize PKCβ isoform expression, RT-PCR analysis using isoform-specific primers was performed. PKCβII transcripts were predominant, whereas PKCβI transcripts were detected at minimal levels (Supplementary Figure S1). To define their functional roles, neutrophils were stimulated with DOG in the presence of isoform-selective PKC inhibitors. Inhibition of PKCβ with LY333531 markedly reduced DOG-induced dextran uptake, whereas pharmacological inhibition with rottlerin, previously described as a PKCδ inhibitor but with known off-target effects [24], had no significant effect under these conditions (Figure 3F–I). Collectively, these data identify PKCβII as the principal downstream effector of DAG–PKC signaling driving macropinocytic uptake in primary human neutrophils.

### 3.4. PKCβ-Dependent Activation of NOX2 Promotes Superoxide Generation in Primary Human Neutrophils

**Figure 4:**
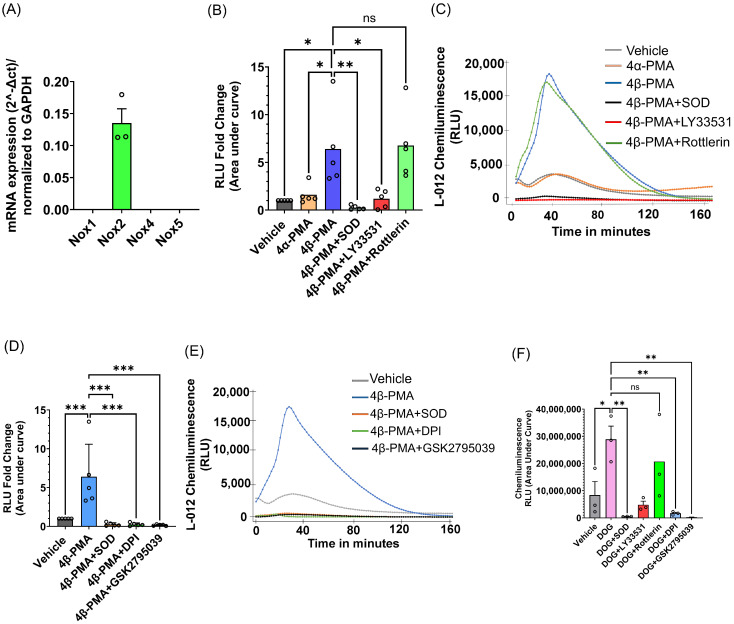
PKCβ-dependent activation of NOX2 promotes superoxide generation in primary human neutrophils. (**A**) qPCR analysis of NOX isoform expression in primary human neutrophils (*n* = 3). (**B**,**C**) Neutrophils were stimulated with vehicle, 4α-PMA, or 4β-PMA in the presence or absence of superoxide dismutase (SOD), the PKCβ inhibitor LY333531, or rottlerin, followed by assessment of ROS production using L-012 chemiluminescence (*n* = 4). (**D**,**E**) Neutrophils were pretreated with diphenyleneiodonium (DPI) or the selective NOX2 inhibitor GSK2795039 prior to 4β-PMA stimulation, and O_2_^•−^ generation was quantified by L-012 chemiluminescence (*n* = 4). (**F**) Neutrophils were stimulated with the DAG analog 1,2-dioctanoyl-sn-glycerol (DOG) in the presence or absence of SOD, LY333531, rottlerin, DPI, or GSK2795039, followed by measurement of ROS production (*n* = 4). Data are presented as means ± SD. Statistical significance was determined using one-way or two-way ANOVA with Tukey’s multiple-comparisons test. ns, not significant; * *p* < 0.05; ** *p* < 0.01; *** *p* < 0.001.

4β-PMA is a DAG mimetic that potently activates PKC signaling and is widely used to stimulate neutrophil activation. PKC activation has been shown to promote phosphorylation of the NOX2 organizer subunit p47*^phox^* [25,26], facilitating assembly of the active NADPH oxidase complex and subsequent superoxide (O_2_^•−^) production. To identify the predominant NADPH oxidase isoform(s) expressed in primary human neutrophils, qPCR analysis was performed. NOX2 was identified as the predominant isoform (Figure 4A), consistent with its established role as the principal ROS-generating NADPH oxidase in neutrophils [17,27]. We next examined whether DAG–PKCβ activation promotes O_2_^•−^ generation using the luminol derivative L-012 (Figure 4B). Stimulation with 4β-PMA induced a rapid and robust increase in chemiluminescence, whereas the inactive stereoisomer 4α-PMA elicited minimal signal. Superoxide dismutase (SOD) markedly reduced the response, confirming that the signal primarily reflects O_2_^•−^ generation. Importantly, inhibition of PKCβ with LY333531 strongly attenuated PMA-induced O_2_^•−^ production, whereas rottlerin had no effect, indicating that ROS generation is largely downstream of PKCβ signaling (Figure 4C). To define the enzymatic source of O_2_^•−^, neutrophils were pretreated with diphenyleneiodonium (DPI), nonselective pan-NOX inhibitor, or the selective NOX2 inhibitor GSK2795039 prior to 4β-PMA stimulation (Figure 4D). Both inhibitors markedly reduced chemiluminescence, confirming that NOX2 is the principal source of PMA-induced O_2_^•−^ production (Figure 4E). We next assessed whether DAG-dependent signaling similarly stimulates O_2_^•−^ production in neutrophils. DOG stimulation induced O_2_^•−^ production, which was abolished by SOD, PKCβ inhibition, and NOX2 blockade, whereas rottlerin had no effect (Figure 4F).

### 3.5. NOX2-Derived Superoxide Anion Stimulates Macropinocytosis in Primary Human Neutrophils

**Figure 5:**
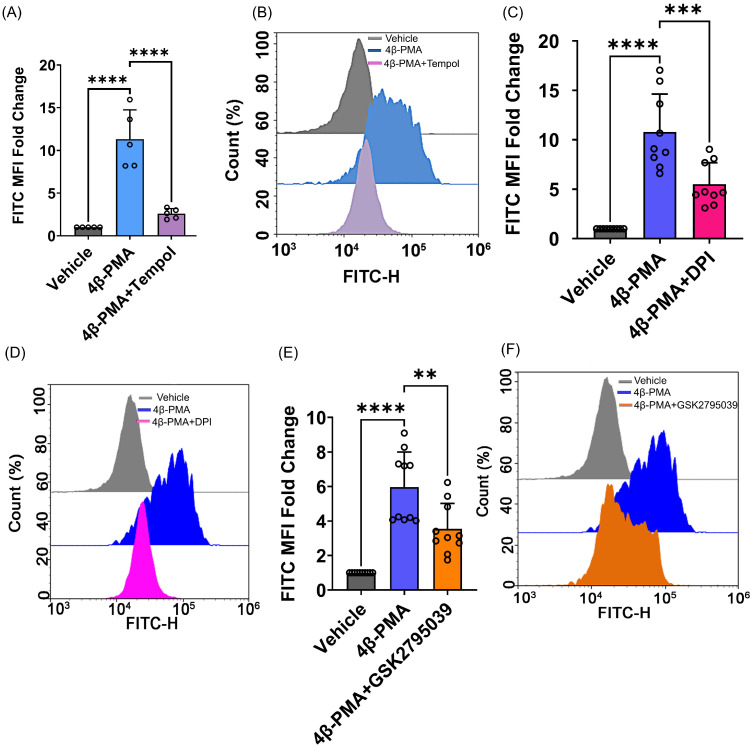
NOX2-derived superoxide anion stimulates macropinocytic uptake in primary human neutrophils (**A**) Primary human neutrophils were treated with vehicle or 4β-PMA (1 μM) in the presence or absence of the membrane-permeable ROS scavenger Tempol, followed by quantification of FITC-dextran uptake by flow cytometry (*n* = 5). (**B**) Representative flow cytometric histograms showing FITC-dextran uptake in vehicle-, 4β-PMA-, and 4β-PMA+Tempol-treated neutrophils. (**C**) Primary human neutrophils were stimulated with vehicle or 4β-PMA in the presence or absence of the NADPH oxidase inhibitor DPI, followed by assessment of FITC-dextran internalization by flow cytometry (*n* = 9). (**D**) Representative flow cytometric histogram plot. (**E**) Primary human neutrophils were stimulated with vehicle or 4β-PMA in the presence or absence of the selective NOX2 inhibitor GSK2795039, followed by quantification of FITC-dextran uptake by flow cytometry (*n* = 9). (**F**) Representative flow cytometric histogram plot. Data are presented as means ± SD. Statistical significance was determined using one-way ANOVA with Tukey’s multiple-comparisons test. Data are presented as means ± SD. Statistical significance was determined using one-way or two-way ANOVA with Tukey’s multiple-comparisons test. ns, not significant; ** *p* < 0.01; *** *p* < 0.001; **** *p* < 0.0001.

Activation of the DAG–PKC signaling pathway is a well-established mechanism regulating assembly and activation of the NOX2-containing NADPH oxidase complex in neutrophils. Upon PKC-dependent phosphorylation of the organizer subunit p47*^phox^*, cytosolic oxidase components translocate to the membrane, resulting in formation of the active NOX2 complex and subsequent superoxide (O_2_^•−^) production. Specifically, we tested the hypotheses that (i) NOX2 activation is required downstream of DAG–PKCβ signaling to stimulate macropinocytosis and (ii) O_2_^•−^ functions as a signaling intermediate in this process. To directly assess the role of ROS, neutrophils were stimulated with 4β-PMA in the presence or absence of Tempol, a membrane-permeable superoxide scavenger and superoxide dismutase mimetic. Consistent with a requirement for ROS signaling, Tempol significantly attenuated 4β-PMA-induced FITC-dextran uptake compared with 4β-PMA alone (Figure 5A). Representative flow cytometric histograms demonstrated a marked leftward shift in FITC fluorescence intensity following Tempol treatment, indicating reduced fluid-phase uptake (Figure 5B). To determine whetherNOX-derived ROS contribute to this response, neutrophils were stimulated with 4β-PMA in the presence of the NADPH oxidase inhibitor DPI. Pharmacological inhibition of NADPH oxidase activity significantly reduced FITC-dextran internalization relative to 4β-PMA-treated cells (Figure 5C). Representative histograms confirmed a substantial reduction in FITC fluorescence intensity in DPI-treated neutrophils (Figure 5D). To specifically examine the contribution of NOX2, neutrophils were treated with the selective NOX2 inhibitor GSK2795039 prior to 4β-PMA stimulation. Inhibition of NOX2 significantly attenuated PMA-induced FITC-dextran uptake compared with 4β-PMA alone (Figure 5E), accompanied by a pronounced leftward shift in FITC fluorescence intensity in representative flow cytometric histograms (Figure 5F). To determine whether extracellular ROS contribute to PMA-induced macropinocytosis, neutrophils were treated with the cell-impermeable ROS scavengers SOD, catalase, or a combination of SOD and catalase prior to PMA stimulation. None of these treatments significantly reduced PMA-induced FITC-dextran uptake, indicating that extracellular superoxide and hydrogen peroxide are not major contributors to PMA-induced macropinocytosis (Supplementary Figure S2). Next, we investigated whether the PKCβ–NOX2 signaling axis regulates GM-CSF-induced macropinocytosis. Neutrophils were stimulated with GM-CSF in the presence of the PKCβ inhibitor LY333531, DPI or the NOX2 inhibitor GSK2795039. Although GM-CSF significantly increased FITC-dextran uptake, inhibition of PKCβ or NOX2 did not attenuate this response, indicating that GM-CSF-induced macropinocytosis is mediated through a PKCβ–NOX2-independent mechanism (Supplementary Figure S3).

## 4. Discussion

Neutrophils are among the earliest immune cells recruited to sites of inflammation, where they orchestrate antimicrobial defense and inflammatory signaling [28]. Although their roles in phagocytosis, degranulation, and ROS-dependent microbial killing are well established, the mechanisms by which neutrophils interrogate and internalize soluble extracellular material remain poorly defined. In particular, macropinocytosis, a conserved actin-driven endocytic pathway mediating bulk uptake of extracellular fluid has been only limitedly described in primary human neutrophils, and its functional significance and signaling mechanisms remain poorly understood. This represents a critical gap, given the emerging appreciation that endocytic trafficking is not merely a housekeeping process but a key regulator of leukocyte activation, metabolic reprogramming, and inflammatory signal integration [29,30]. Defining whether neutrophils possess a regulated macropinocytic program, and how it is coupled to inflammatory signaling pathways, is therefore essential for understanding how these cells sense and adapt to complex tissue microenvironments.

A central finding of this study is that primary human neutrophils exhibit a rapid, inducible, and actin-dependent macropinocytic response. 4β-PMA stimulation triggered membrane ruffling, formation of cup-like structures, and uptake of high-molecular-weight dextran. This response was inhibited by EIPA and cytochalasin D, consistent with canonical requirements for Na^+^/H^+^ exchanger activity and actin cytoskeletal remodeling. Together, these data are consistent with induction of macropinocytic uptake in neutrophils rather than nonspecific dye accumulation or passive uptake mechanisms. Macropinocytosis is a multistep and highly dynamic process involving coordinated membrane ruffling, macropinosome formation, and vesicular maturation [31]. Consistent with this temporal organization, neutrophil macropinocytic activity was transient and tightly regulated, peaking at approximately 60 min of stimulation before declining. The transient nature of this response suggests that macropinocytosis is closely coupled to early activation signaling events rather than a sustained uptake pathway in neutrophils. Importantly, the use of Oregon Green dextran confirmed that the observed uptake reflects true fluid-phase internalization and is not attributable to pH-dependent changes in fluorophore intensity resulting from endosomal acidification.

Our data further demonstrates that neutrophil macropinocytosis is stimulus-selective and context-dependent. Among a range of growth factors and cytokines, GM-CSF and HGF were the only mediators that significantly enhanced macropinocytic uptake in an EIPA-sensitive manner. In contrast, PDGF, CXCL12, TSP1, and EGF had no detectable effect. GM-CSF is a well-established neutrophil priming and survival factor, while HGF has been implicated in tissue remodeling and repair processes. These findings suggest that neutrophil macropinocytosis is preferentially engaged in inflammatory microenvironments enriched in survival and tissue-regenerative cues, rather than being broadly responsive to chemotactic or growth factor stimulation.

Mechanistically, we identify DAG-dependent PKC signaling as a proximal regulator of neutrophil macropinocytosis, with PKCβ serving as the dominant functional isoform. Pharmacological activation of DAG signaling using DOG was sufficient to induce dextran uptake, while inhibition with EIPA demonstrated that this response is mediated by macropinocytosis. Consistently, 4β-PMA induced robust uptake, whereas the inactive stereoisomer 4α-PMA was ineffective. Isoform-selective pharmacology further demonstrated that PKCβ, but not PKCδ, is required for DAG-dependent macropinocytic activation. In addition, qRT-PCR analysis using isoform-specific primers identified PKCβII as the predominant PKCβ isoform expressed in human neutrophils, supporting its potential role in regulating macropinocytosis. Taken together, these findings establish PKCβ as a key signaling molecule linking lipid second messengers to actin-driven membrane remodeling in neutrophils.

A major and previously unrecognized finding of this study is the coupling of PKCβ signaling to NOX2-dependent ROS production in the regulation of macropinocytosis. Neutrophils predominantly express NOX2, which mediates the oxidative burst via O_2_^•−^ generation. We show that activation of DAG–PKCβ signaling robustly induces NOX2-dependent O_2_^•−^ production, which is abolished by pharmacological inhibition of PKCβ or NOX2. Importantly, both pharmacological NADPH oxidase inhibition with DPI and selective NOX2 inhibition with GSK2795039 reduced ROS production and macropinocytic uptake in neutrophils, indicating that NOX2-derived ROS contribute to the regulation of this pathway. Interestingly, GM-CSF-induced neutrophil macropinocytosis was not inhibited by either PKCβ or NOX2 inhibition, suggesting that distinct signaling mechanisms regulate macropinocytosis depending on the stimulus. A limitation of this study is the use of pharmacological approaches to investigate the PKCβ–NOX2 signaling axis. Future studies using genetic models will provide complementary validation of the contribution of this pathway to neutrophil macropinocytosis. In addition, live-cell or fluorescence imaging analyses were not performed to directly visualize macropinocytosis in primary human neutrophils.

Beyond their antimicrobial role, ROS are increasingly recognized as signaling molecules that regulate cytoskeletal dynamics and membrane trafficking [32,33]. In this context, our findings support a model in which NOX2-derived O_2_^•−^ acts downstream of PKCβ to promote actin-dependent membrane ruffling and macropinocytic cup formation. Consistent with this interpretation, scavenging ROS attenuated macropinocytic uptake, indicating that oxidant production is not merely a byproduct of neutrophil activation but an active regulator of macropinocytosis.

Together, these findings establish macropinocytosis as a regulated and stimulus-dependent endocytic pathway in primary human neutrophils and identify a DAG–PKCβ–NOX2–ROS signaling axis that governs this process. This pathway expands the functional repertoire of neutrophils beyond classical phagocytosis and oxidative killing, highlighting a previously unrecognized role for redox signaling in macropinocytic uptake. In a broader physiological context, neutrophils are continuously exposed to cytokines, growth factors, and tissue-derived mediators within inflamed environments. Macropinocytosis may therefore represent an adaptive mechanism by which neutrophils sample extracellular fluid, integrate inflammatory cues, and modulate functional responses during tissue injury and repair. In conclusion, we propose a model in which DAG-dependent activation of PKCβ initiates NOX2-derived ROS production, which in turn promotes actin-driven macropinocytic uptake in human neutrophils. Previous studies have demonstrated that NOX2-derived ROS regulate multiple redox-sensitive signaling pathways involved in actin remodeling. For example, ROS can reversibly oxidize and inhibit protein tyrosine phosphatases, thereby prolonging kinase signaling pathways that regulate actin cytoskeletal remodeling and membrane dynamics required for macropinocytosis [34]. ROS also modulate Rho family GTPase signaling, including Rac1, Cdc42, and RhoA, which coordinate actin polymerization and membrane protrusion [35]. In addition, ROS may also regulate actin cytoskeletal dynamics through redox-sensitive signaling pathways, including the LIMK/cofilin axis and cortactin, thereby coordinating the balance between actin polymerization, filament stabilization, and turnover required for membrane ruffling and macropinosome formation [36]. Whether intracellular NOX2-derived ROS engage these or other redox-sensitive signaling networks to promote macropinocytic membrane ruffling and cup formation in primary human neutrophils remains to be determined. Future studies should identify the specific redox-sensitive effectors that couple NOX2-derived ROS to cytoskeletal remodeling and macropinocytosis. In vivo studies will be essential to establish the physiological and pathological roles of neutrophil macropinocytosis in immune function and disease.

## Data Availability

The original contributions presented in this study are included in the article/Supplementary Materials. Further inquiries can be directed to the corresponding author.

## Supplementary Materials

The following supporting information can be downloaded at https://www.mdpi.com/article/10.3390/antiox15070904/s1, Figure S1: Relative mRNA expression of PKCβI and PKCβII isoforms in primary human neutrophils. Relative transcript levels of PKCβI and PKCβII were determined in isolated primary human neutrophils by quantitative RT-PCR. Gene expression was normalized to GAPDH, which was used as the internal reference control. Data are presented as mean ± SD; Figure S2: Effect of non-permeable reactive oxygen species scavengers on PMA-induced macropinocytosis in primary human neutrophils. Primary human neutrophils were pretreated with superoxide dismutase (SOD), catalase, or a combination of SOD and catalase prior to stimulation with 4β-PMA, and macropinocytosis was quantified by measuring FITC-dextran uptake using flow cytometry. These non-permeable ROS scavengers were used to assess the contribution of extracellular ROS to PMA-induced macropinocytosis. Data are presented as mean ± SEM. Statistical significance was determined by one-way ANOVA followed by Tukey’s multiple comparisons test; Figure S3: GM-CSF-induced macropinocytosis is independent of PKCβ–NOX2 signaling. Primary human neutrophils were pretreated with LY333531, GSK2795039, or DPI prior to stimulation with GM-CSF, and macropinocytosis was assessed by quantifying FITC-dextran uptake using flow cytometry. Data are presented as mean ± SD. Statistical analysis was performed by one-way ANOVA with Tukey’s multiple comparisons test.
