# Supplementary material for: Mechanistic Insights into Redox-Dependent Macropinocytosis in Primary Human Neutrophils
Source: Antioxidants (Basel). 2026 Jul 21;15(7):904. doi: 10.3390/antiox15070904 (PMC13406137; doi:10.3390/antiox15070904)
Supplement: Supplementary file 1 [file antioxidants-15-00904-s001.zip › antioxidants-4401191-supplementary.pdf]

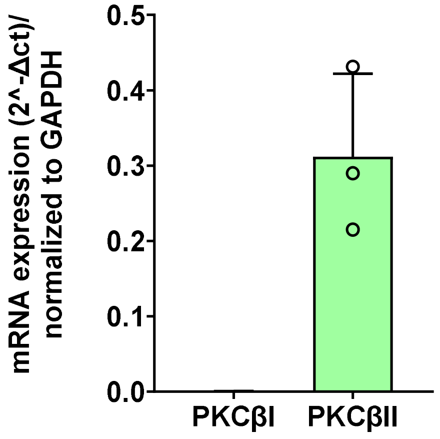

Supplementary Figure S1. Relative mRNA expression of PKCβI and PKCβII isoforms in primary human neutrophils. Relative transcript levels of PKCβI and PKCβII were determined in isolated primary human neutrophils by quantitative RT-PCR. Gene expression was normalized to GAPDH, which was used as the internal reference control. Data are presented as mean ± SD.

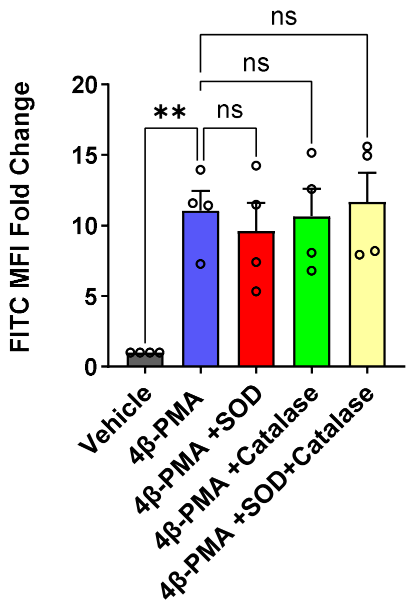

Supplementary Figure S2. Effect of non-permeable reactive oxygen species scavengers on PMA-induced macropinocytosis in primary human neutrophils. Primary human neutrophils were pretreated with superoxide dismutase (SOD), catalase, or a combination of SOD and catalase prior to stimulation with 4β-PMA, and macropinocytosis was quantified by measuring FITC-dextran uptake using flow cytometry. These non-permeable ROS scavengers were used to assess the contribution of extracellular ROS to PMA-induced macropinocytosis. Data are presented as

mean ± SEM. Statistical significance was determined by one-way ANOVA followed by Tukey’s multiple comparisons test.

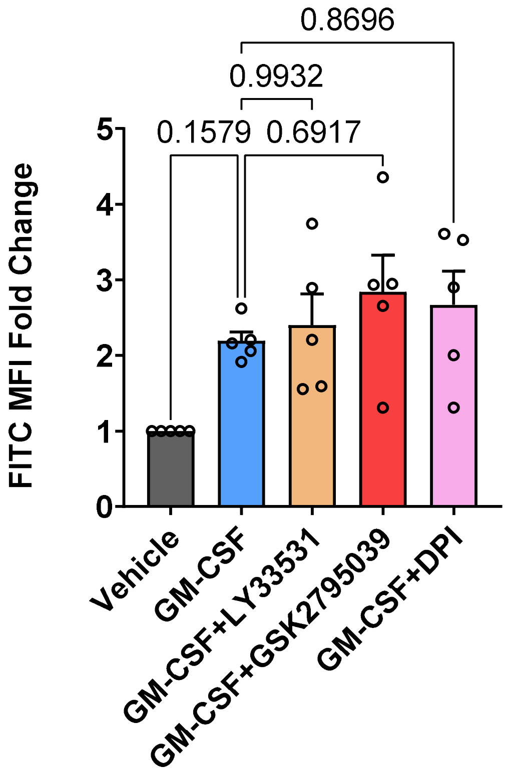

Supplementary Figure S3. GM-CSF-induced macropinocytosis is independent of PKCβ–NOX2 signaling. Primary human neutrophils were pretreated with LY333531, GSK2795039, or DPI prior to stimulation with GM-CSF, and macropinocytosis was assessed by quantifying FITC-dextran uptake using flow cytometry. Data are presented as mean ± SD. Statistical analysis was performed by one-way ANOVA with Tukey's multiple comparisons test.
